# Iron: an essential micronutrient for the legume-rhizobium symbiosis

**DOI:** 10.3389/fpls.2013.00359

**Published:** 2013-09-13

**Authors:** Ella M. Brear, David A. Day, Penelope M. C. Smith

**Affiliations:** ^1^School of Biological Sciences, The University of SydneySydney, NSW, Australia; ^2^School of Biological Sciences, Flinders UniversityBedford Park, Adelaide, SA, Australia

**Keywords:** legume–rhizobium symbiosis, nitrogen fixation, nodule, iron, symbiosome, bacteroid, symbiosome membrane

## Abstract

Legumes, which develop a symbiosis with nitrogen-fixing bacteria, have an increased demand for iron. Iron is required for the synthesis of iron-containing proteins in the host, including the highly abundant leghemoglobin, and in bacteroids for nitrogenase and cytochromes of the electron transport chain. Deficiencies in iron can affect initiation and development of the nodule. Within root cells, iron is chelated with organic acids such as citrate and nicotianamine and distributed to other parts of the plant. Transport to the nitrogen-fixing bacteroids in infected cells of nodules is more complicated. Formation of the symbiosis results in bacteroids internalized within root cortical cells of the legume where they are surrounded by a plant-derived membrane termed the symbiosome membrane (SM). This membrane forms an interface that regulates nutrient supply to the bacteroid. Consequently, iron must cross this membrane before being supplied to the bacteroid. Iron is transported across the SM as both ferric and ferrous iron. However, uptake of Fe(II) by both the symbiosome and bacteroid is faster than Fe(III) uptake. Members of more than one protein family may be responsible for Fe(II) transport across the SM. The only Fe(II) transporter in nodules characterized to date is GmDMT1 (*Glycine max* divalent metal transporter 1), which is located on the SM in soybean. Like the root plasma membrane, the SM has ferric iron reductase activity. The protein responsible has not been identified but is predicted to reduce ferric iron accumulated in the symbiosome space prior to uptake by the bacteroid. With the recent publication of a number of legume genomes including *Medicago truncatula* and *G. max,* a large number of additional candidate transport proteins have been identified. Members of the NRAMP (natural resistance-associated macrophage protein), YSL (yellow stripe-like), VIT (vacuolar iron transporter), and ZIP (Zrt-, Irt-like protein) transport families show enhanced expression in nodules and are expected to play a role in the transport of iron and other metals across symbiotic membranes.

## INTRODUCTION

All plants require the micronutrient iron for optimum growth. However, legumes, which develop symbiotic relationships with nitrogen-fixing bacteria, have an increased demand for the micronutrient (Tang et al., 1990a). Both the plant and bacteria individually have an innate requirement, but it is also essential for the establishment, development, and function of the symbiosis (O’Hara, 2001). This review will focus on the role of iron in the legume–rhizobium symbiosis, specifically iron movement within the symbiotic organ, the nodule. We will also describe how the analysis of legume genomes and transcriptomes will enhance the identification of iron transporters in the nodule.

## THE IMPORTANCE OF LEGUMES

Symbiotic nitrogen fixation (SNF) by rhizobia, housed within legume nodules, converts abundant but biologically unavailable atmospheric nitrogen to ammonia. Thus the ability of legumes to obtain fixed nitrogen from the bacteroid offers a growth advantage as soil nitrogen often limits plant growth (Graham and Vance, 2003). Not only is the symbiosis beneficial to the legume, SNF introduces approximately 40 million tonnes of nitrogen into agricultural soils each year (Herridge et al., 2008). This injection of nitrogen can be utilized by subsequent crops and reduces reliance on application of synthetic nitrogen fertilizer for enhanced crop yields.

## THE DEVELOPMENT OF THE LEGUME–RHIZOBIUM SYMBIOSIS

Development of the symbiosis results in the production of a new plant organ, the root nodule, where SNF occurs. The induction of nodule organogenesis involves a signaling exchange between free-living soil bacteria and the legume host (Popp and Ott, 2011). This signaling dialog produces specificity to the interaction and ultimately results in attachment of rhizobia to the legume root hair cells and prepares the legume for infection (Schultze and Kondorosi, 1998).

Rhizobia attached to a root hair are transported toward root cortical cells, within an infection thread before being released into root cortical cells where they are surrounded by a plant-derived membrane, the symbiosome membrane (SM) that separates them from the plant cell cytoplasm (Whitehead and Day, 1997). Rhizobia once differentiated into their symbiotic nitrogen fixing form are called bacteroids. The bacteroid, SM and the space surrounding the bacteroid, the symbiosome space, together comprise the symbiosome.

Rhizobia are released into root cortical cells from the infection thread via a process that shares similarities to exocytosis (Limpens et al., 2009; Ivanov et al., 2012). During release the rhizobia become encapsulated by the infection thread membrane, which is continuous with the plant plasma membrane (Whitehead and Day, 1997). To accommodate bacterial infection and proliferation, it is estimated that 21,500 μm^2^ of SM is synthesized per infected cell (Roth and Stacey, 1989). Following initial formation, the composition of the SM reflects its plasma membrane origin, but modifications to its composition are continually made for the membranes specialized new role (Verma et al., 1978; Fortin et al., 1985). This modification includes synthesis and incorporation of new lipids and proteins, and is mediated by the secretory pathway (Catalano et al., 2004). The importance of the secretory pathway to nodule formation in *Medicago truncatula* is emphasized by secretory proteins making up 62% of all proteins expressed in nodules (Maunoury et al., 2010). The identity of the SM can be described as a mosaic that changes throughout development (Limpens et al., 2009). Following symbiosome formation and until senescence the SM is labeled with the known plasma membrane SNARE protein SYP132 (Limpens et al., 2009). Later, during symbiosome differentiation to senescence the late endosomal marker, Rab7 (Limpens et al., 2009), labels the SM. Vacuolar SNAREs are also acquired on the SM during senescence (Limpens et al., 2009).

**FIGURE 1:**
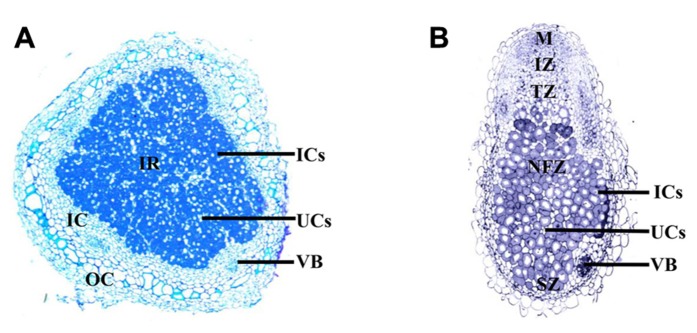
**Longitudinal cross section of the determinant soybean nodule (A), compared to a longitudinal cross section of the indeterminant *M. truncatula *nodule (B)**. The determinant soybean nodule consists of a central infected region (IR), composed of infected cells (ICs; dark) dispersed between uninfected cells (UCs; light); surrounding the IR is the inner cortex (IC), which includes the vascular bundles surrounded by parenchyma cells; the outer cortex (OC), encapsulates the nodule. The meristem of the indeterminant *M. truncatula* nodule remains active throughout nodule development, allowing continual growth. As a result the nodule is divided into sequential zones of development, including the meristem (M); invasion zone (IZ), where rhizobia infect divided cortical cells; a transition zone (TZ), composed of infected cells containing differentiating bacteria; a nitrogen fixation zone (NFZ) and a zone of senescence (SZ) close to the root. Images courtesy of Aleksandr Gavrin.

The mature nodule is composed of the central infection zone, containing infected and uninfected cells, surrounded by layers of cells termed the cortex (Udvardi and Poole, 2013). Metabolites are transported to the nodule through the vasculature, which terminates in the cortex (Udvardi and Poole, 2013). Nodules can be divided into two types, determinant and indeterminant (**Figure 1**). Soybean and *Lotus japonicus* produce determinant nodules, which are spherical and contain bacteroids all at approximately the same developmental stage (Udvardi and Poole, 2013). Whereas indeterminant nodules, which develop on *M. truncatula*, *Pisum sativum*, and clover, are characterized by elongated or branched structures with meristems that remain throughout the life of the nodule. Unlike determinant nodules, which contain an infected region that matures and senesces together, indeterminant nodules are segmented into developmental zones a meristematic zone, an invasion zone where rhizobia are first released, a transition zone where bacteroids differentiate, a nitrogen fixation zone and a zone of senescence closest to the root (Udvardi and Poole, 2013).

The symbiosome can be thought of as a plant organelle with a specialized function for nitrogen fixation. Thus the symbiosome is expected to have unique properties that may change throughout nodule development to meet the requirements of differentiating bacteroids, nitrogen fixation and senescence (Whitehead and Day, 1997). Within the symbiosome, the bacteroid is reliant on the legume for the supply of a carbon source and all the other nutrients essential for bacterial metabolism and nitrogen fixation, apart from nitrogen itself (Udvardi and Day, 1997). All nutrients transported to the bacteroid must first cross the SM, allowing the legume to remain in control of the symbiosis.

## IRON DEFICIENCY AND THE LEGUME–RHIZOBIUM SYMBIOSIS

Legumes involved in a nitrogen-fixing symbiosis have a greater requirement for iron (Tang et al., 1990a). The rate of nitrogen fixation in *Phaseolus vulgaris* L. nodules is positively correlated with increasing nodule iron concentrations (Slatni et al., 2008). Under iron deficient conditions soybean and peanut with active nodules have a heightened response to iron deficiency (O’Hara et al., 1988; Terry et al., 1991). Iron stress induces the secretion of H^+^ into the rhizosphere and the activity of Fe(III) reductase in soybean, to enhance iron uptake from the soil (Terry et al., 1991). Lupins require a greater supply of iron when relying on SNF for the supply of nitrogen when compared to plants grown with nitrogen fertilizer (Tang et al., 1990b).

Iron deficiency can affect both the legume host and the rhizobia individually or can have a direct affect on their interaction. All legumes are affected by iron deficiency, but the effect on the symbiosis varies between legume species. Studies on iron deficiency and nodule formation have been conducted for peanut, chickpea, lupin, lentil, soybean, and French bean (Tang et al., 1990b). Iron deficiency affected either nodule initiation or later development. When *Lupinus angustifolius* L. is grown under iron deficiency, fewer nodules form, indicating an effect on nodule initiation (Tang et al., 1990a). In contrast iron deficiency does not affect the initiation of nodules in peanuts, common bean and soybean, but rather affects later nodule development (O’Hara et al., 1988; Soerensen et al., 1988; Slatni et al., 2011). Peanuts grown under iron deficiency have lower concentrations of leghemoglobin, a delayed onset of nitrogen fixation and up to 215 times fewer bacteroids within the infected region (O’Hara et al., 1988), indicating an effect on the differentiation of rhizobia and the development of the resulting bacteroids.

When *Lupinus angustifolius *L*.* plants were grown in a split root system so that the effect of reduced metabolite supply from the shoot could be distinguished from the direct affects of iron deficiency on the symbiosis, iron was not translocated from an uninoculated root exposed to sufficient iron to the roots exposed to iron deficiency and there was no affect of foliar application of iron on nodule development, suggesting that the signaling of iron deficiency is not systemic (Tang et al., 1990b). However, in the same experiment peanut showed enhanced nodule initiation, development, and nitrogen fixation following application of foliar iron compared to an iron deficient control (O’Hara et al., 1988).

## REQUIREMENT FOR IRON IN THE SYMBIOSIS

The requirement for iron by legumes with an active symbiosis is large because many symbiotic proteins incorporate iron. Iron is required by the very numerous bacteroids for the synthesis of the nitrogen-fixing enzyme, nitrogenase, as well as cytochromes, ferredoxin, and hydrogenase (Guerinot, 1991; Delgado et al., 1998; O’Hara, 2001; Dixon and Kahn, 2004; Peters and Szilagyi, 2006). This requirement for iron by the symbiosis is highlighted by the proportion of iron within the nodule compared to other plant organs. At nodule maturity soybean nodules have the highest iron concentration, approximately 44% of the iron within soybean plants is present in the nodule compared to 31% in leaves, 7% in seed, and 5% in roots (Burton et al., 1998). At seed maturity, the seed has the highest iron concentration of all organs approximately 35% compared to 27% in the nodule, 23% in leaves, 9% in roots, and 3% in the stem (Burton et al., 1998).

Nitrogenase is a metalloenzyme, which catalyses the conversion of atmospheric dinitrogen to ammonia. Iron is essential in the two components that make up nitrogenase. The iron protein is the smaller component, which is reduced and provides electrons to the molybdenum-iron protein, a larger, heterotetrameric component that contains the catalytic site (Dixon and Kahn, 2004). At the catalytic site, dinitrogen binds and is reduced (Peters and Szilagyi, 2006). Both the iron protein and the molybdenum-iron protein are sensitive to oxygen.

Other iron-containing proteins essential for the symbiosis include ferredoxin, a non-heme protein, involved in transferring electrons and reducing the iron component of nitrogenase (Dixon and Kahn, 2004), and cytochrome components of the bacterial respiratory electron transport chain, essential for providing the energy for nitrogen fixation (Delgado et al., 1998).

There is a conflicting requirement for oxygen by the bacteroid. Nitrogenase is extremely sensitive to oxygen, thus the nodule must maintain low oxygen concentrations while maintaining oxygen supply for bacterial metabolism (Appleby, 1984). As well as an oxygen diffusion barrier in the cortex, infected cells synthesize leghemoglobin to bind oxygen and facilitate diffusion to the bacteroids, while maintaining oxygen concentrations at microaerobic levels for both respiration and nitrogen fixation (Appleby, 1984). Leghemoglobin is present within the cytoplasm of infected cells, at a concentration of approximately 3 mM (Bergersen and Appleby, 1981). Whether leghemoglobin is present within the symbiosome space is controversial (Appleby, 1984). However, if present it is found at low concentrations, approximately 200–500 μM (Bergersen and Appleby, 1981). The apoprotein and heme moiety, both components of leghemoglobin are synthesized by the plant (O’Brian, 1996). Iron is incorporated into the protoporphyrin ring by iron chelatase during the final stage of the tetrapyrrole biosynthetic pathway, resulting in the formation of protoheme (Vavilin and Vermaas, 2002). This protoheme is then incorporated into the apoprotein synthesized in the plant cytoplasm (Verma et al., 1979). An estimated 24% of soluble iron within the nodule is present within leghemoglobin (Ragland and Theil, 1993), thus iron plays an important role in maintaining the nodule environment for the symbiosis.

## IRON SUPPLY TO THE NODULE

Iron is transported throughout the plant within the xylem, where it is maintained as a ferric citrate complex due to the low pH of the xylem (Cline et al., 1982). There is evidence for transport of ferric citrate in a number of plant species including the legume, soybean (Tiffin, 1970; Lopez-Millan et al., 2000). Tri-iron (III), tri-citrate (Fe_3_Cit_3_) is the main iron citrate species transported in tomato xylem exudates (Rellan-Alvarez et al., 2010). As well as iron movement within the xylem, nodules may also take up ferrous iron directly, as there is a ferric chelate reductase on the surface of *Phaseolus vulgaris* L. nodules (Slatni et al., 2009). Analysis of an iron efficient common bean variety with antibodies raised against a H^+^-ATPase and *Arabidopsis* IRT1 (iron transporter 1), suggests that immunologically related proteins are present in nodule cortex cells in response to iron deficiency and that direct uptake of iron from the rhizosphere may complement supply from the plant when iron availability is limiting (Slatni et al., 2012). However, when the localization of iron was observed within the indeterminant *M. truncatula* nodule, no iron was localized at the epidermis of the nodule (Rodriguez-Haas et al., 2013), suggesting that direct uptake of iron from the rhizosphere by the nodule is not the main route of iron acquisition.

## IRON CONCENTRATIONS ACROSS NODULE DEVELOPMENT

Throughout nodule development, the concentration and distribution of iron within the nodule fluctuates as the role of the symbiotic organ changes over time. Rodriguez-Haas et al. (2013) recently monitored iron distribution in indeterminant *M. truncatula* nodules using synchrotron-based X-ray fluorescence and their results enhance previously proposed theories about iron movement within the nodule.

The nodule meristem is characterized by low concentrations of iron (Rodriguez-Haas et al., 2013). During the early stages of soybean nodule development the concentration of the iron storage protein ferritin increases, reaching maximum concentration 12 days after inoculation (DAI) with rhizobia (Ragland and Theil, 1993). Within the nodule, ferritin accumulates in both infected and uninfected cells (Lucas et al., 1998). This accumulation is proposed to concentrate iron ready for incorporation into nitrogenase and leghemoglobin, beginning around 12 DAI (Ragland and Theil, 1993). Iron concentration within the nodule increases greatly between 12 and 15 DAI, remaining constant until 36 DAI (Ragland and Theil, 1993). Iron is abundant within the apoplast of zone II, while in zone III, the region of nitrogen fixation, iron becomes incorporated into infected cells (Rodriguez-Haas et al., 2013). Many of the iron-containing symbiotic proteins are synthesized within the bacteroid so following iron incorporation into infected cells iron must then transverse both the symbiosome and bacteroid membrane.

Iron concentrations within the determinant soybean nodule began to decrease 39 DAI (Burton et al., 1998). During senescence, at approximately 77 DAI, the decline in iron concentration is met with an increase in ferritin, which is thought to complex iron produced from the breakdown of leghemoglobin and nitrogenase, ready for remobilization to the seed (Burton et al., 1998; Lucas et al., 1998). Iron remobilization from the senescing *M. truncatula* nodule is supported by accumulation of iron around vessels near the senescing zone (Rodriguez-Haas et al., 2013).

## IRON MOVEMENT WITHIN THE NODULE

Within the nodule, iron must be directed to the cells and organelles that synthesize iron-containing proteins. In the case of nitrogenase and cytochromes of the bacteroid respiratory chain, iron must firstly enter the infected cells of the nodule and then transverse the SM before being taken up by the bacteroid from the symbiosome space. Iron is also required in the cytoplasm of infected cells for incorporation into leghemoglobin, as well as in very numerous mitochondria that line the periphery of these cells (Wittenberg et al., 1996). The movement of iron throughout the plant relies on the strict control of redox state and chelate formation, to avoid iron toxicity and precipitation (Kobayashi and Nishizawa, 2012). Evidence for how iron is transported across the numerous nodule membranes to the bacteroid is limited but what is known is presented below.

### IRON UNLOADING FROM THE XYLEM

Although iron unloading from xylem vessels has not been directly studied in nodules, it may have similarities to iron transport from xylem to leaf mesophyll cells. Iron (III)-citrate is transported to the shoot in xylem vessels and is released into the apoplast (Brüggemann et al., 1993).

### FROM VASCULATURE TO INFECTION ZONE

**FIGURE 2:**
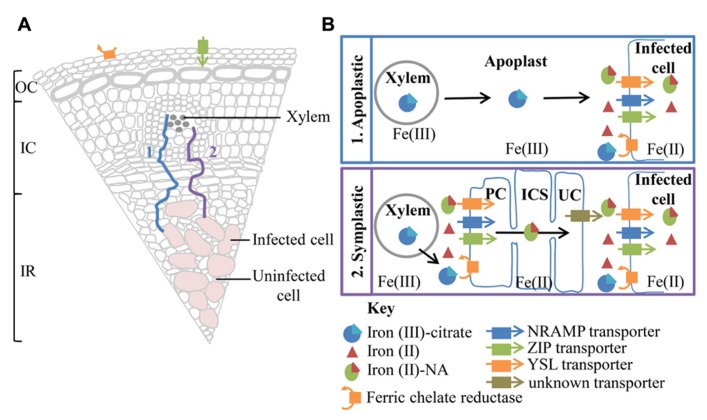
**A summary of possible transport pathways for iron from the xylem to the infected cell (A) and a more detailed depiction of the iron species and transporters involved (B). (A)** The majority of iron entering the nodule is derived from iron citrate, translocated within the xylem. However, the presence of a ferric chelate reductase and a ZIP family, transporter related to *A. thaliana* IRT1, on the surface of the nodule, suggests a mechanism for direct iron uptake from the soil. The nodule is divided into three main sections the outer cortex (OC), inner cortex (IC), and infected region (IR). The xylem and infected cells are separated by a number of cell layers. Movement of iron to the infected cell could be either via an apoplastic (1) or symplastic (2) route. **(B)** Apoplastic transport involves the unloading of ferric citrate from the xylem into the apoplast. Ferric-citrate diffuses through the apoplast toward the infected cell, stabilized by the low pH. Uptake by the infected cells involve the reduction of ferric iron to ferrous iron. The resulting ferrous iron could then be imported into the infected cell by a member of the NRAMP or ZIP transporter family. Alternatively ferrous iron could chelate to nicotianamine (NA) and be imported into the infected cell via a member of the YSL transporter family. A symplastic path to the infected cell would require the initial reduction of ferric citrate by a ferric chelate reductase, localized on cells surrounding the xylem, possibly a pericycle cell (PC). Ferrous iron could then be imported into the cell via similar transport mechanisms utilized for uptake into the infected cell, highlighted above. Iron could move symplastically through the pericycle cells, inner cortex cells (ICS) and uninfected cells (UC) as a ferrous iron-NA chelate. Uninfected cells neighboring the infected cells possibly efflux ferrous iron-Na or dissociated ferrous iron. Now in the apoplast the ferrous iron could be oxidized and chelated to citrate to increase solubility. However, for import into the infected cell ferric iron must be reduced to ferrous iron.

A number of cell layers, including one or two layers of distributing, boundary and pericycle cells, separate the vascular bundle from the infected zone in soybean nodules (Guinel, 2009). Little is known about iron movement between the xylem and infected cells, both symplastic and apoplastic routes are postulated (**Figure 2**).

The presence of plasmodesmata connections between all cell layers from pericycle cells, adjacent to xylem, to infected cells suggests a possible route for symplastic transport to infected cells in soybean nodules (Brown et al., 1995). Symplastic transport within the nodule would require the reduction of iron (III) and dissociation from citrate (Brüggemann et al., 1993). The resulting iron (II) then could be chelated to nicotianamine (NA) and imported into the cell via members of the yellow stripe-like (YSL) transporter family or taken up directly as ferrous iron via members of the natural resistance-associated macrophage protein (NRAMP) or ZIP (Zrt-, Irt-like protein) transporter families prior to chelation to NA (Kobayashi and Nishizawa, 2012). The iron(II)-NA chelate could then be readily transferred via symplastic route. Despite the presence of plasmodesmata connections between cells spanning from xylem to infected cell, the movement of symplastic and apoplastic dyes within *M. truncatula* nodules suggests a barrier to symplastic continuity (Bederska et al., 2012). There appears to be a requirement for localized apoplastic transport within the pericycle, surrounding the xylem and prior to uptake into the infected cell (Bederska et al., 2012).

Alternatively Fe(III)-citrate, could be transported apoplastically toward the infected region. The slightly acidic pH of the apoplast would promote the oxidation of iron and the formation of Fe(III)-citrate. In *M. truncatula* nodules, Rodriguez-Haas et al. (2013), using synchrotron-based X-ray fluorescence, observed a thread-like distribution of iron around cells in the nodule parenchyma and particularly in zone II, suggesting iron is moving in the apoplast. Confirmation of the route of iron movement between the vasculature and infection zone will enable predictions about transport proteins involved in iron transport toward and into symbiosomes.

### IRON TRANSPORT INTO THE INFECTED CELL

**FIGURE 3:**
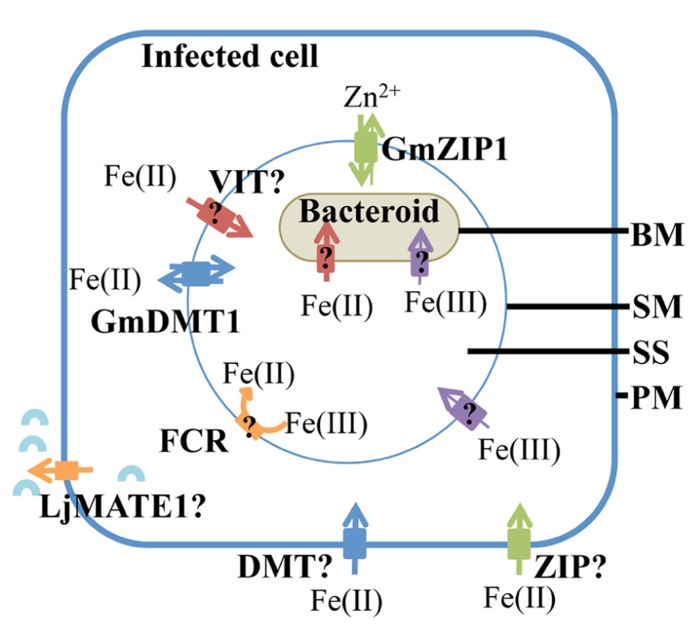
**Iron uptake into the infected cell and transport into the symbiosome.** Ferrous iron transport into the infected cells, across the plasma membrane (PM) could me mediated by transporters belonging to the NRAMP, ZIP, or YSL family. A citrate effluxer, LjMATE1 is expressed in infected cells, it is predicted to release citrate (blue arc) into the apoplast surrounding infected cells to aid in iron uptake. It is likely to make iron in the apoplast more soluble. Both ferrous and ferric iron are transported across the symbiosome membrane (SM) and bacteroid membrane (BM). However, ferrous iron is taken up more rapidly. A ferric iron transporter, GmDMT1, is present on the SM, but it may not be the only ferrous iron transporter on the SM. Members of the VIT and ZIP transporter families are possible ferrous iron transporters on the SM. Although ferric iron is thought to cross the SM, no plant transporters are known to transport iron-citrate. Ferric chelate reductase activity (FCR) is present on the SM, however, the orientation on the SM has not been confirmed. Within the symbiosome space (SS) ferric iron could chelate siderophores produced by the bacteroid. Ferrous and ferric iron are transported across the bacteroid membrane (BM), but the transporters responsible have not been identified.

Iron transport into *Lotus japonicus* infected cells is enhanced by efflux of citrate via LjMATE1 (*Lotus japonicus* multidrug and toxic compound extrusion 1; Takanashi et al., 2013; **Figure 3**), suggesting that it occurs as a ferric citrate complex. LjMATE1 is expressed exclusively in infected cells early in development and catalyses efflux of citrate when expressed in *Xenopus* oocytes (Takanashi et al., 2013). When expression of LjMATE1 was reduced by RNAi, nitrogenase activity and leghemoglobin concentration were significantly decreased compared to control nodules (Takanashi et al., 2013), apparently as a result of less iron in infected cells. This decrease in leghemoglobin synthesis and nitrogenase activity was accompanied by increased concentrations of iron at the nodule–root junction and the vascular bundle of nodules. The early expression of LjMATE1 and its importance to concentrating iron within the infected region of nodules, suggests that citrate efflux into the apoplast of the infected region is important for iron import into these cells. In this context, LjMATE1 may play a similar role to FRD3, a MATE family member in *Arabidopsis* (Roschzttardtz et al., 2011). *frd3* mutants show iron deficiency in leaves even though iron uptake from the soil is constitutively active. FRD3 is a citrate effluxer and is thought to release citrate into the apoplast, to chelate iron and make it more soluble, to enable transport into the cytoplasm (Roschzttardtz et al., 2011). The protein/s responsible for transport across the plasma membrane of infected cells in soybean and other legumes are not known. A member of the Zrt-, Irt-like protein (ZIP) or NRAMP family may be involved but this would require reduction of Fe(III) by a ferric-chelate reductase before uptake (**Figure 3**). As iron is likely to be imported into infected cells from the apoplast in *M. truncatula* (Rodriguez-Haas et al., 2013), it is likely that similar transporter families are involved in uptake in indeterminant nodules. Transcriptome studies of *M. truncatula* suggest that expression of members of these families is enhanced in nodules (Benedito et al., 2008).

### IRON TRANSPORT ACROSS THE SYMBIOSOME MEMBRANE

Iron can cross the SM as both Fe(III) and Fe(II) (Moreau et al., 1995, 1998; LeVier et al., 1996; **Figure 3**). This was indicated by uptake of radiolabeled ferric and ferrous iron by isolated soybean symbiosomes. Inhibition of ferrous iron transport into symbiosomes by Cu(II) suggests that the protein responsible may not be specific for iron. The uptake of ferrous iron [Fe(II)] was faster than that of ferric iron [Fe(III); Moreau et al., 1998). Whether uptake of ferrous iron is favored by the symbiosome within the natural nodule environment is yet to be determined and will most likely depend on the concentrations of Fe(II) and Fe(III) in the infected cell cytosol. Unlike the soil environment, where iron is found in an oxidized state, the nodule cytosol provides conditions for maintaining iron in its reduced ferrous state (Moreau et al., 1995). This environment is created by the slightly acidic pH, microaerobic environment and the abundance of reducing molecules such as ascorbate and glutathione in the nodule infected cells. Ferric chelate reductase activity has been identified on the SM (LeVier et al., 1996). Initially it was thought that the reductase activity occurred on the cytoplasmic side of the SM, where Fe(III)-citrate, which is present at high concentrations within the nodule, is reduced before uptake into the symbiosome (LeVier et al., 1996). However, Moreau et al. (1998) postulated that the reductase activity was present within the symbiosome space based on the discovery of ferrous iron transport across the SM and the orientation of the plasma membrane reductase (outside the plasma membrane). The protein responsible for the ferric chelate reductase activity has not been identified to date, but two out of nine genes encoding ferric chelate reductase proteins (Glyma15g13090 and Glyma16g03770) are expressed in nodules at a higher level compared to root tissue (Libault et al., 2010; Severin et al., 2010; see **Table 1**). Due to their nodule expression, these proteins are potential candidates for the ferric chelate reductase activity of the SM. It will be interesting to determine their localization and role in iron movement within the nodule. The current model of iron transport across the SM postulates that both ferrous and ferric iron can be transported across the SM, although a transporter for Fe(III)-citrate has not been characterized in dicot plants (**Figure 3**). According to this model, ferric iron is reduced to ferrous iron within the symbiosome space by the SM ferric chelate reductase. The resulting ferrous iron could either be transported out of the symbiosome space into the bacteroid or into the infected cell cytosol (Moreau et al., 1998).

**Table 1:** Ferric chelate reductase family members encoded in the Soybean genome.

	Transcriptome				TMD	Homology to AtFRO2 (% similarity)
	Severin et al. (2010)		Libault et al. (2010)			
	Root	Nodule	Root	Nodule		
Glyma05g02600	1	0	0	0	8	30.57
Glyma07g07380	44	0	366	0	8	56.55
Glyma09g02170	0	0	3	0	9	29.93
Glyma10g37600	15	0	529	0	9	50.00
Glyma10g37610	1	1	16	0	9	49.79
Glyma15g13090	0	0	5	11	9	29.66
Glyma16g03770	0	11	0	93	9	55.43
Glyma17g09260	2	0	17	0	9	29.82
Glyma18g47060	0	0	0	0	9	54.81

*A comparison of each reductase's expression in root and nodule tissue from two soybean transcriptome studies is presented (Libault etal., 2010; Severin etal., 2010). A prediction of the number of transmembrane domains (TMD) was analyzed by SOSUI (). Homology to AtFRO2, a characterized, ferric chelate reductase present in the outer root layers of *A. thaliana*, was calculated by a clustalW alignment.*

A ferrous iron transporter, *Glycine max* divalent metal transporter 1 (GmDMT1), with homology to the NRAMP transporter family, has been identified on the SM of soybean (Kaiser et al., 2003; **Figure 3**). GmDMT1 was able to complement the yeast iron transport mutant *fet3fet4* (Kaiser et al., 2003). Rates of Fe(II) uptake by the yeast were similar to the kinetics observed for ferrous uptake into symbiosomes (Moreau et al., 1998). The ability of GmDMT1 to partially complement the zinc uptake mutant (ZHY3) and the ability of excess manganese to interrupt the uptake of ferrous iron, suggests that GmDMT1 is not specific for iron transport. Given ferrous iron uptake was inhibited by copper II in assays with isolated symbiosomes (Moreau et al., 1998) it would be interesting to determine if GmDMT1 also transports copper II.

Although there is evidence that ferrous iron is transported across the SM and that GmDMT1 is present on the SM, this does not prove GmDMT1’s role in iron uptake into the symbiosome. The ability of GmDMT1 to complement a yeast mutant for iron transport on the plasma membrane, *fet3fet4,* suggests involvement in import of iron into the cell. However, the direction of transport into the symbiosome is similar to transport across the vacuolar membrane and therefore would be similar to efflux from the cell. Thus the orientation of GmDMT1 on the SM must be determined and its importance in iron uptake investigated, perhaps through RNAi disruption.

Members of the ZIP family of transporters, GmZIP1, are involved in iron transport in some plants and GmZIP1 has been detected on the SM (Moreau et al., 2002; **Figure 3**). However, there is no evidence so far that GmZIP1 transports iron and yeast complementation suggests a role in zinc transport (Moreau et al., 2002). The ZIP family of transporters and their possible role in iron transport within the nodule will be discussed later.

### IRON AND THE SYMBIOSOME SPACE

It appears that the majority of iron transported into symbiosomes is not directly incorporated into the bacteroid (LeVier et al., 1996). Rather, the symbiosome space appears to be a storage site for iron within the nodule. The concentration of non-heme iron within the symbiosome space is estimated to be approximately 0.5–2.5 mM, and is thought to be complexed with siderophores derived from the bacteroid (Wittenberg et al., 1996), this represents approximately 7–20% of total non-heme iron extracted from whole nodules. The symbiosome space has a lower pH than the plant cytosol owing to the action of both symbiotic partners (Pierre et al., 2013). An H^+^-ATPase localized to the SM pumps protons from the plant cytoplasm into the symbiosome space, while the bacteroid also contributes protons to the symbiosome space through the action of the electron transport chain (Udvardi and Day, 1997). Pierre et al. (2013) estimated the pH of the symbiosome space to range between 4.5 and 5 using acidotropic probes. The low pH of the symbiosome space would promote the stabilization of ferric chelates such as ferric citrate (Cline et al., 1982).

### IRON UPTAKE BY THE BACTEROID

The majority of the symbiotically important iron-containing proteins are synthesized within the bacteroid. Consequently iron must be taken up by the bacteroid from the symbiosome space. Regulation of iron uptake has been extensively studied in free-living rhizobia. However, whether bacteroids within the symbiosome use the same iron uptake mechanisms as their free-living counterparts is still to be determined (Fabiano and O’Brian, 2012). A number of transcriptome studies comparing gene expression between free-living rhizobia and symbiotic bacteroids at different developmental stages have been conducted for a range of rhizobial strains (Barnett et al., 2004; Becker et al., 2004; Capela et al., 2006; Chang et al., 2007), but detailed comparisons of iron uptake into free-living bacteria and nitrogen-fixing bacteroids are lacking. Regulation of iron uptake by free-living, culture grown rhizobia has been extensively studied and role of iron responsive transcriptional regulators, such as IrrA and rirA, and the genes that they control under both iron deficient and sufficient conditions determined (Viguier et al., 2005; Todd et al., 2006). Many of the genes controlled by the regulators include genes for siderophore production, heme biosynthesis, and transporters such as a ferric siderophore ATP-binding cassette (ABC) transporter (Viguier et al., 2005; Todd et al., 2006).

Free-living rhizobia have a number of mechanisms to take up and compete for scarce iron from the soil. These include the release of ferric iron chelating siderophores, the reduction of ferric iron to ferrous iron followed by uptake of the resulting ferric iron, and the ability to utilize iron from heme compounds (Fabiano and O’Brian, 2012). Evidence for similar mechanisms of uptake into bacteroids is discussed below.

#### Ferrous iron

Isolated bacteroids can take up ferrous iron (LeVier et al., 1996; Moreau et al., 1998; **Figure 3**) and at a faster rate than ferric iron. A homolog of the transporter involved in ferrous iron uptake, *Escherichia coli, *FeoB**(Hantke, 2003) has been identified in *Bradyrhizobium japonicum* but has not been characterized (Fabiano and O’Brian, 2012).

#### Ferric iron chelates

One of the most common mechanisms for iron uptake uses low molecular weight, high affinity ferric iron ligands called siderophores. The formation of the siderophore-ferric iron chelate solubilizes ferric iron, allowing uptake. Rhizobia can either take up siderophores synthesized *de novo* and released or can scavenge siderophores produced by other soil microbes. The type of siderophore synthesized is not a characteristic of rhizobial strain (Guerinot, 1994), but fall into three major classes of siderophores including α-hydroxycarboxylates, catecholates, and hydroxamates (Miethke and Marahiel, 2007). Siderophore–ferric iron complexes are actively transported across the outer and cytoplasmic membrane of gram negative rhizobia. Ferric iron–siderophore complexes bind to TonB-dependent receptors on the outer membrane and are actively transported into the pericycle following activation by a cytoplasmic membrane complex (TonB–ExbBD), which couples the outer membrane to the proton motive force of the cytoplasmic membrane (Lim, 2010). Following release into the pericycle, the siderophore complex is taken up into the cytoplasm by ABC importers (Faraldo-Gomez and Sansom, 2003). Within the cytoplasm, the siderophore complex is dissociated by the reduction of ferric iron to ferrous iron (Matzanke et al., 2004). A ferric reductase has been identified in *B. japonicum *(Small and O’Brian, 2011).

Under iron limiting conditions, free-living rhizobia express TonB-dependent receptors after activation by the iron response regulator (Irr; Small et al., 2009). In contrast, active transport of siderophores by bacteroids appears to be unnecessary for the symbiosis. Evidence for this includes down regulation of expression of siderophore and heme TonB-dependent receptors and TonB itself, as well as Irr (Yeoman et al., 2000; Chang et al., 2007; Small et al., 2009) and ABC transporters (Barnett et al., 2004) in bacteroids. Mutations in ABC transporters, TonB-dependent receptors and TonB itself, have no affect on the function of the established symbiosis (Lynch et al., 2001; Nienaber et al., 2001; Wexler et al., 2001). This suggests that bacteroids do not require high affinity siderophore uptake to obtain iron during the symbiosis. However, *Sinorhizobium meliloti* have increased nodule occupancy under iron limiting conditions, compared to mutant strains with impaired siderophore uptake systems (Battistoni et al., 2002b). The ability of rhizobia to take up iron chelated to siderophores appears to provide a competitive advantage and may affect the effectiveness of the resulting symbiosis.

Although the proteins essential for siderophore uptake in rhizobia do not appear to be essential for the symbiosis, iron-binding chromophores have been identified within the symbiosome space (Wittenberg et al., 1996). Wittenberg et al. (1996) isolated the siderophore complexes from nodules infected with three different bradyrhizobial strains. The size and optical spectra of the isolated siderophore complexes differed between strains and it was hypothesized that the siderophores were of bacteroid origin.

Another ferric-chelating compound utilized by some rhizobia for iron uptake is citrate. Citrate has a lower affinity for iron than classic siderophores and is released by cells experiencing iron limitation. A strain of *B. japonicum *(61A152), a symbiotic partner of soybean, secretes citrate under iron limitation and was able to take up radiolabeled iron citrate (Guerinot et al., 1990). The mechanism of ferric citrate uptake by rhizobia has not been characterized but it possibly shares similarities with the ferric citrate uptake mechanisms of *E. coli, *another gram negative bacterium*. E. coli* utilize a similar mechanism for uptake of ferric citrate and ferric-siderophore chelates, requiring a TonB-dependent receptor on the outer membrane and a member of the ABC transporter family for transport across the cytoplasmic membrane (Enz et al., 2000).

Like their free-living counterparts, isolated *B. japonicum* bacteroids can take up ferric citrate complexes labeled with ^59^Fe (Moreau et al., 1995). However, uptake of Fe(III)-chelates across the bacteroid membrane is very slow compared with uptake of ferrous iron (LeVier et al., 1996; Moreau et al., 1998). If ferric chelates are not the major source of iron transported into bacteroids, it raises the issue of why bacteroids produce siderophores. It is not known whether bacteroids experience iron limitation in the symbiotic environment. However, the reduced expression of proteins involved in siderophore synthesis and ferric siderophore uptake suggests not only that ferric iron chelates are not the major form of iron transported to the bacteroid, but also that the bacteroid, does not experience iron stress like free-living rhizobia. Ferric iron may have a greater importance as the iron storage form within the symbiosome space (LeVier et al., 1996), where it is captured by siderophores after transport across the SM so it remains sequestered within the symbiosome space (Wittenberg et al., 1996). The stored ferric iron could then be reduced to ferrous iron prior to uptake by the bacteroid (LeVier et al., 1996).

#### Heme uptake

As mentioned earlier, leghemoglobin is abundant within the nodule. Like pathogenic bacteria that infect animals, free-living rhizobia are able to utilize leghemoglobin, heme, and hemoglobin as iron sources when iron is limiting (Noya et al., 1997). Bacteroids do not have contact with the pool of leghemoglobin found within infected cells (Wittenberg et al., 1996) and therefore it is probably not a major source of iron during symbiosis. However, leghemoglobin may be of importance during nodule senescence (Noya et al., 1997) because the nodule cytosol becomes acidic and this promotes the dissociation of heme from leghemoglobin (Herrada et al., 1993). Also, the membranes within the nodule including the SM degrade and rhizobia could utilize this pool of newly available heme or the heme could be released into the rhizosphere following degradation of the nodule (Noya et al., 1997). The mechanism used by rhizobia to take up heme appears to be similar to transport mechanisms used for siderophore uptake (Noya et al., 1997). Further evidence for the ability of rhizobia to utilize heme as an iron source is shown by the discovery of a putative high affinity heme-binding outer membrane protein (Battistoni et al., 2002a). However, like mechanisms for siderophore uptake, mutations in heme transport proteins do not affect the symbiosis (Nienaber et al., 2001;Wexler et al., 2001), suggesting heme uptake is not essential for iron supply to the bacteroid.

### SENESCENCE AND REMOBILIZATION OF IRON IN THE NODULE

During nodule senescence, membranes degrade and iron is released, inhibiting nitrogen fixation and thereby triggering further senescence. Iron present within leghemoglobin plays a key role in promoting senescence of the nodule. When the nodule begins to senesce, the nodule cytosol becomes more acidic, promoting autoxidation of leghemoglobin and production of superoxide anions and hydrogen peroxide (Herrada et al., 1993). Hydrogen peroxide is known to dissociate iron from leghemoglobin and this free iron can degrade membranes within the nodule (Herrada et al., 1993). This is a problem for indeterminant nodules because within the one nodule there are regions undergoing senescence, while other regions are actively fixing nitrogen. It has been postulated that the increase in ferritin observed within younger infected regions close to senescing zones might restrict the spread of iron-induced senescence and prolong nitrogen fixation (Strozycki et al., 2007). This differs from determinant nodules where the whole nodule senesces at the same time. However, the observation that ferritin increases in the cortex of senescing lupin nodules may also be to contain iron spread during senescence (Lucas et al., 1998).

At the time of nodule senescence, formation of the seed becomes the priority for the legume and the high concentrations of iron in the nodule provide a ready supply of iron for the seed (Burton et al., 1998). The available iron may include iron from leghemoglobin, which is known to decrease in the nodule at senescence (Puppo et al., 1991). During the period of seed filling nodules were shown to lose between 40 and 58% of radiolabeled iron, thus nodules may contribute to a large proportion of seed iron if all this iron is transported to the seed (Burton et al., 1998). This iron may be transported to the seed as a NA-chelate in the phloem (Curie et al., 2009).

A protein potentially involved in the redistribution of nodule iron is *L. japonicas* NA synthase 2 (LjNAS2; Hakoyama et al., 2009). NA synthase catalyses the formation of NA, a phytosiderophore precursor that is present in all plants and forms complexes with a range of metals including Fe(II) and Fe(III) (Curie et al., 2009). LjNAS2 is expressed in nodule vascular bundles, is nodule specific and its expression reaches a maximum at 24 DAI (Hakoyama et al., 2009). Suppression of LjNAS2 expression specifically by RNAi silencing did not have an affect on nitrogen fixation, suggesting that LjNAS2 is not involved in iron supply to the nodule (Hakoyama et al., 2009). This phenotype could possibly indicate the redundancy of LjNAS2. However, only one other NAS, LjNAS1, is encoded in the *L. japonicas* genome (Hakoyama et al., 2009). LjNAS1 has 62.3% amino acid homology to LjNAS2, but unlike LjNAS2, LjNAS1 is expressed predominantly in cotyledons, leaves, and stems, with expression in nodules very low (Hakoyama et al., 2009). Due to the low nodule expression of LjNAS1, the phenotype observed due to suppression of LjNAS2, suggests LjNAS2’s role is not redundant. Hakoyama et al. (2009) hypothesize that LjNAS2 may play a role in remobilization of iron from the nodule at senescence and this is supported by the late expression of LjNAS2 during nodule development. Future studies observing LjNAS2 knockdown phenotypes during nodule senesces and seed maturation may enable the role of LjNAS2 to be further dissected. If iron is remobilized from nodules, chelated to NA, members of the YSL family may play a role. A number of which are expressed in nodules and will be discussed later (Libault et al., 2010; Severin et al., 2010).

## POTENTIAL IRON TRANSPORTERS WITHIN THE NODULE

There are clear roles for iron transport within the nodules and we can predict where they should be localized, but only one nodule iron transporter, DMT1, has been functionally characterized and it is localized to the SM. Transcriptome analysis of the recently available legume genomes allows us to identify genes with nodule enhanced expression and, together with our knowledge of transporter function in our systems, to predict proteins with important roles in iron transport in the nodule. Movement of iron into infected cells, for example, is likely to occur via a transporter involved in uptake into the cell, such as ZIP and NRAMP family members. Movement of iron into the symbiosome, on the other hand, could occur via an efflux transporter, such as the vacuolar iron transporter (VIT) family. Members of the YSL family are candidates for remobilization of iron from the nodule and possibly for ferrous iron chelate transport into the symbiosome. Here we will summarize the transcriptome information from soybean to highlight genes encoding possible iron transport proteins important in nodules.

### THE ZIP FAMILY

Members of the ZIP transporter family can transport cadmium, zinc, copper, manganese, and iron in a diverse range of organisms (Hall and Guerinot, 2007). Members of this transporter family include the yeast zinc transporters Zrt1p and Zrt2p and the plant iron transporters AtIRT1, MtZIP6, and PsRIT1 (Vert et al., 2002; Cohen et al., 2004; Lopez-Millan et al., 2004). MtZIP6 can transport both iron and zinc (Lopez-Millan et al., 2004). The direction of transport is generally into the cellular cytoplasm, including transport across the plasma membrane into the cytosol and transport across organelle membranes into the cytosol (Hall and Guerinot, 2007). On this basis, ZIP transporters could be involved in transport of iron into infected cells or out of the SM. However, the first ZIP family member to be characterized in soybean, GmZIP1, appears to transport zinc in the opposite direction to all other ZIP family members because GmZIP1-specific antibodies inhibited zinc transport into isolated symbiosomes (Moreau et al., 2002). This suggests that the orientation on the SM may not follow transporter orientation seen for the PM or organelles, or that GmZIP1 allows bidirectional transport, because when it is expressed in yeast, it catalyzed import of zinc across the PM.

The* G. max* genome encodes 19 ZIP family members, with all but six expressed in nodules (**Table 2**). Five of these, Glyma14g37560, Glyma20g06210 (GmZIP1), Glyma15g41620, Glyma13g10790, and Glyma06g05460, have increased expression in nodules compared with other plant tissues. Analysis of nodule microsomal fractions using antibodies directed against AtIRT suggested at least three members of the ZIP family were expressed in nodules, although only one protein band could be identified in SM preparations, presumably corresponding to GmZIP1 (Moreau et al., 2002). This supports a role for ZIP proteins on membranes other than the SM in nodules and these might be responsible for transport of iron.

**Table 2:** Expression of the ZRT, IRT-like (ZIP) family transporters encoded in the soybean genome.

	Transcriptome				TMD	Homology to MtZIP6 (% similarity)	Homology to AtIRT1 (% similarity)
	Severin et al. (2010)		Libault et al. (2010)				
	Root	Nodule	Root	Nodule			
Glyma02g13950	0	0	0	0	8	48	53
Glyma04g05410	2	1	0	0	4	42	39
Glyma06g05460	217	83	128	332	6	41	42
Glyma07g34930	40	0	38	0	8	73	59
Glyma08g17530	19	5	4	13	8	45	44
Glyma08g44010	8	5	19	2	9	19	23
Glyma11g27900	3	5	19	6	9	40	40
Glyma13g10780	0	0	0	0	-	44	40
Glyma13g10790	447	36	55	79	4	48	45
Glyma13g41330	46	6	247	1	9	20	21
Glyma14g37560	1	42	9	69	8	41	39
Glyma15g04090	59	24	141	43	9	18	20
Glyma15g04100	1	0	1	0	9	18	20
Glyma15g41620	25	16	4	42	8	46	44
Glyma17g34660	11	10	26	16	9	43	39
Glyma18g06740	4	7	19	14	8	41	39
Glyma18g08760	4	7	11	1	9	19	22
Glyma20g02770	97	0	255	0	8	74	61
Glyma20g06210	29	36	6	48	8	48	44

*A comparison of each ZIP transporters expression in root and nodule tissue from two soybean transcriptome studies is presented (Libault etal., 2010; Severin etal., 2010). A prediction of the number of transmembrane domains (TMD) for each protein was analyzed using SOSUI (). Homology to characterized ferrous iron transporters of the ZIP family, MtZIP6 and AtIRT1 was calculated by a clustalW alignment.*

### NRAMP FAMILY OF TRANSPORTERS

The NRAMP transporter family are present in bacteria, plants, fungi, and mammals and are involved in general metal ion transport (including ferrous iron), driven by a proton gradient (Nevo and Nelson, 2006). In plants, NRAMP proteins can transport iron (Lanquar et al., 2005), manganese (Cailliatte et al., 2010; Lanquar et al., 2010; Sasaki et al., 2012), cobalt (Cailliatte et al., 2010), cadmium (Sasaki et al., 2012), and aluminum (Xia et al., 2010), and often have broad specificity. AtNRAMP3 and AtNRAMP4 are H^+^ metal symporters responsible for iron and Mn mobilization from the vacuole (Lanquar et al., 2005, 2010). Many of the PM localized transporters are involved in transport of metals other than iron (Cailliatte et al., 2010; Sasaki et al., 2012), but peanut AhNRAMP1 is likely to be involved in iron acquisition from the soil (Xiong et al., 2012). Plant NRAMP proteins are generally involved in import into the cytoplasm, although there is some argument over the direction of transport of the mammalian NRAMP1 that suggests it could act as an exporter.

In the soybean genome, 17 genes are predicted to encode members of the NRAMP/DMT protein family (**Table 3**). Four of these are homologs of EIN2, a regulator of the ethylene-signaling pathway in *Arabidopsis* (Alonso et al., 1999) and are unlikely to be involved in metal ion transport. Ten of the classical NRAMP genes are expressed in nodules, with expression of three genes, Glyma04g04660, Glyma06g04720, and Glyma17g18010, enhanced in nodules compared with roots (**Table 3**). Glyma17g18010 corresponds to GmDMT1, the ferrous iron transporter localized on the SM (Kaiser et al., 2003). The three proteins have higher similarity to AtNRAMP3 than any other soybean family members (**Table 3**).

**Table 3:** Expression of members of the NRAMP transporter family encoded in the soybean genome.

	Transcriptome				TMD	Homology to AtNRAMP3 (% similarity)
	Severin et al. (2010)		Libault et al. (2010)			
	Root	Nodule	Root	Nodule		
Glyma01g39790	3	1	9	3	10	77
Glyma03g33850	6	3	29	26	11	18
Glyma04g04660	5	79	15	94	11	70
Glyma05g21780	7	3	23	7	9	75
Glyma06g04720	3	35	11	114	11	71
Glyma06g12190	82	4	263	4	11	36
Glyma07g02680	3	0	6	1	11	37
Glyma07g06490	0	0	0	0	11	64
Glyma08g23320	16	8	35	6	11	37
Glyma10g06610	7	6	39	40	11	20
Glyma11g05500	5	1	14	6	10	78
Glyma13g20810	11	5	53	42	11	19
Glyma13g44710	2	0	1	0	11	34
Glyma15g00590	2	1	4	3	12	35
Glyma16g03090	0	0	0	0	11	65
Glyma17g18010	14	32	63	63	9	75

*A comparison of each NRAMP transporter's expression in root and nodule tissue from two soybean transcriptome studies is presented (Libault etal., 2010; Severin etal., 2010). A prediction of the number of transmembrane domains (TMD) for each protein was analyzed using SOSUI (). Homology to a characterized ferrous iron transporter of the NRAMP family, AtNRAMP3, was calculated by a clustalW alignment.*

Based on the characterization of known NRAMP/DMT proteins, NRAMP/DMT homologs could be involved in metal ion transport across a number of membranes within the nodule. Similarity to AtNRAMP3 and 4 suggests they maybe localized to the vacuole or SM where they could re-mobilize stored iron. Since there is debate about the direction of transport of NRAMP1 in macrophages it is possible that NRAMP proteins expressed in nodules like GmDMT1 could participate in remobilization of iron from the symbiosome or uptake into the symbiosome (**Figure 3**). Other NRAMP proteins could be present on the plasma membrane of infected cells and mediate uptake into the infected cell (**Figure 3**).

### THE VACUOLAR IRON TRANSPORTER FAMILY

Since uptake into the symbiosome involves efflux from the plant cell, we could predict that iron transporters present on the vacuolar membrane in other organisms could play a role in iron uptake into the symbiosome. Members of the VIT family are involved in the uptake of Fe(II) into the vacuole for storage. In yeast, CCC1 (Lapinskas et al., 1996; Li et al., 2001) and in *Arabidopsis* VIT1 (Kim et al., 2006), fulfill this role. In plants the VIT family includes two different groups, those with close homology to VIT1 and those with similarity to Nodulin21 from soybean (Delauney et al., 1990). Iron transport activity has not been proved for members of the Nodulin21 group although mutation of one member in *Lotus japonicus,* LjSEN1, blocks nitrogen fixation (Hakoyama et al., 2012), and some of the *Arabidopsis* members are regulated by iron availability (Gollhofer et al., 2011).

*LjSEN1* is expressed specifically in infected cells of the nodule and the protein it encodes is proposed to be a ferrous iron transporter based on its distant homology to AtVIT1 (Kim et al., 2006) and CCC1 (Li et al., 2001). The development of symbiosomes is affected in *sen1* nodules. Infected cells at 8 DAI had multiple vacuoles and large symbiosomes with a seemingly large symbiosome space surrounding the bacteroids. Expression of *LjSEN1* in *S. cerevisiae* did not increase iron concentrations within transformed yeast cells (Hakoyama et al., 2012). However, this may be attributed to the expected localization of LjSEN1 to vacuoles within the yeast cell, rather than the cell membrane. Complementation studies expressing AtVIT1 in *Δccc1* yeast mutants provided evidence that AtVIT1 localizes to the vacuolar membrane in yeast. Thus LjSEN1 would also be predicted to localize to the vacuolar membrane in yeast and would not mediate iron uptake into yeast. It will be interesting to find the location of SEN1 in the nodule and to test for ferrous iron transport in *Δccc1* yeast mutants (Hakoyama et al., 2012).

The soybean genome encodes 20 members of the VIT family. Only two are closely related to VIT1. Expression of two of the Nodulin21-like genes, Glyma05g25010 and Glyma08g08120, is very high in nodules and not detected in any other tissue (**Table 4**). They have greater similarity to LjSEN1 than to AtVIT1 (**Table 4**). Due to the importance of LjSEN1 to the symbiosis, Glyma05g25010 and Glyma08g08120 are interesting candidates as possible essential iron transporters for the symbiosis.

**Table 4:** Expression of members of the vacuolar iron transporter (VIT) family encoded in the soybean genome.

	Transcriptome				TMD	Homology to AtVit1 (% similarity)	Homology to LjSEN1 (% similarity)
	Severin et al. (2010)		Libault et al. (2010)				
	Root	Nodule	Root	Nodule			
Glyma01g36530	0	0	0	0	4	21	52
Glyma02g09110	18	1	2	19	4	22	51
Glyma05g24980	0	0	0	2	5	19	52
Glyma05g24990	0	0	0	1	5	20	54
Glyma05g25000	0	1	8	0	4	20	65
Glyma05g25010	0	761	16	2134	5	19	65
Glyma05g34430	5	8	3	6	5	81	22
Glyma08g05230	5	3	10	2	5	82	22
Glyma08g08070	10	0	0	8	4	12	36
Glyma08g08090	6	1	0	17	4	19	54
Glyma08g08100	3	0	0	8	5	19	54
Glyma08g08110	0	0	3	0	4	21	63
Glyma08g08120	0	192	3	801	4	21	62
Glyma08g19390	3	0	1	7	4	19	52
Glyma10g37030	1	0	0	1	4	24	57
Glyma11g08830	0	0	0	1	5	20	54
Glyma15g05610	0	0	0	1	3	19	50
Glyma16g28340	21	4	9	61	4	22	52
Glyma18g46245	0	0	–	–	5	22	17
Glyma20g30580	0	3	–	–	4	21	55

*A comparison of each transporter's expression in root and nodule tissue from two soybean transcriptome studies is presented (Libault etal., 2010; Severin etal., 2010). A prediction of the number of transmembrane domains (TMD) each protein has was analyzed by SOSUI (). Homology to two VIT family members, AtVIT1, a characterized ferrous iron transporter and LjSEN1, which is essential for nitrogen fixation in L. japonicus, were calculated by a clustalW alignment.*

### THE YSL FAMILY

The YSL family of transporters forms a distinct group in the oligopeptide (OPT) superfamily with less than 20% similarity to other members (Curie et al., 2009; Ueno et al., 2009). The founding member of the YSL transporter family is ZmYS1, which is a symporter coupled to proton transport, and its expression is enhanced under iron deficiency (Roberts et al., 2004; Schaaf et al., 2004). YS1 is able to transport Fe(III) complexed to the phytosiderophores deoxymugineic acid (DMA) and mugeneic acid (MA), as well as Fe(II) and Fe(III) complexed to NA, although the Fe(II) complex is transported more readily (Roberts et al., 2004; Schaaf et al., 2004). In monocots, the family mediates the uptake of Fe(III)-phytosiderophore complexes from the rhizosphere (Ueno et al., 2009). Dicots also contain members of the YSL family, but, because they do not take up siderophore complexes from the soil, it is thought that they specialize in long distance transport of Fe(II)-NA within the plant (Ueno et al., 2009). YSL family members can also transport Cu (Roberts et al., 2004), Ni (Gendre et al., 2007), and Mn (Sasaki et al., 2011) complexed to PS. Most YSL transporters characterized are localized to the plasma membrane and are involved in uptake of metals. AtYSL4 and 6 are the exceptions as they are localized to the chloroplast membrane. However, their direction of transport – out of the chloroplast to reduce iron toxicity – is analogous to that of the plasma membrane transporters (Divol et al., 2013).

Iron remobilization from the nodule will involve transporters that are expressed later in nodule development, during seed formation. The identification of a NA synthase in *Lotus japonicus* nodules and its expression later in nodule development suggests that iron is redistributed from the nodule chelated to NA (Hakoyama et al., 2009). This makes YSL family members candidates for iron remobilization from the nodule during senescence.

Fifteen YSL family members are encoded in the soybean genome. Of these, Glyma11g31870 has essentially nodule specific expression while expression of eight other members of the family has been detected in nodules (**Table 5**). In soybean, the transcriptome has been studied at only one time-point – that of mature N-fixing nodules – and so genes with enhanced expression during nodule senescence may not be obvious.

**Table 5:** Expression of the YSL family transporters encoded in the soybean genome.

	Transcriptome				TMD	Homology to AtYSL1 (% similarity)
	Severin et al. (2010)		Libault et al. (2010)			
	Root	Nodule	Root	Nodule		
Glyma04g41020.1	6	4	15	18	12	64
Glyma06g13820.1	5	5	22	37	14	64
Glyma09g29410.1	2	1	7	2	14	53
Glyma09g41800.1	0	0	0	0	10	45
Glyma10g31610.1	0	0	1	0	13	71
Glyma11g31870.1	0	25	2	7	12	52
Glyma13g10410.1	0	1	3	2	11	68
Glyma16g05850.1	21	6	91	47	12	54
Glyma16g33840.1	14	4	123	99	13	53
Glyma17g26520.1	0	0	1	2	15	62
Glyma19g26500.1	27	8	90	58	13	54
Glyma20g00690.1	0	0	0	0	14	48
Glyma20g00700.1	0	0	0	0	11	48
Glyma20g16600.1	0	0	0	0	12	73
Glyma20g35980.1	0	2	6	7	13	71

*A comparison of each YSL transporter's expression in root and nodule tissue from two soybean transcriptome studies is presented (Libault etal., 2010; Severin etal., 2010). A prediction of the number of transmembrane domains (TMD) for each protein was analyzed by SOSUI (). Homology to AtYSL1, a characterized ferrous iron transporter of the YSL family, was calculated by a clustalW alignment.*

## CONCLUDING REMARKS AND FUTURE DIRECTIONS

Iron transport in the roots and nodules of symbiotic legumes is clearly very complex, involving many cell types, some unique to nodules, and transport both into and out of cells and organelles. Transport across the specialized SM is especially intriguing and of special significance to nitrogen fixation. Given the importance of iron to the symbiosis and the symbiosis to sustainable agriculture, it is important that we understand the processes involved in iron acquisition, storage, and mobilization.

Our knowledge of transporters in nodules to date is derived from classical genetic approaches including screening for sequence homology to known iron transporters (for example *GmZIP1* and *GmDMT1; *Moreau et al., 2002; Kaiser et al., 2003) or the identification of genes involved in certain mutant phenotypes (e.g., LjSEN1; Hakoyama et al., 2012). Recent advances in genome sequencing, transcriptomics, and proteomics, open new avenues for identifying transport functions. In particular, the large scale sequencing of *M. truncatula*, *Lotus japonicus*, and *G. max *genomes has resulted in an explosion in the list of genes encoding membrane proteins (Benedito et al., 2010), many of them highly expressed in nodules and some of them probable iron transporters. The challenge is to functionally characterize these transporters and to identify their location and roles within nodules.

## Conflict of Interest Statement

The authors declare that the research was conducted in the absence of any commercial or financial relationships that could be construed as a potential conflict of interest.
